# Challenges in measurement of linkage following HIV self‐testing: examples from the STAR Project

**DOI:** 10.1002/jia2.25238

**Published:** 2019-03-25

**Authors:** Melissa Neuman, Miriam Taegtmeyer, Karin Hatzold, Cheryl C Johnson, Helen A Weiss, Katherine Fielding

**Affiliations:** ^1^ MRC Tropical Epidemiology Group Faculty of Epidemiology and Population Health London School of Hygiene and Tropical Medicine London United Kingdom; ^2^ Liverpool School of Tropical Medicine Liverpool United Kingdom; ^3^ Population Services International Johannesburg South Africa; ^4^ Department of HIV and Global Hepatitis World Health Organization Geneva Switzerland

**Keywords:** HIV testing, HIV self‐testing, linkage to care, research methods

Knowledge of HIV status through HIV testing constitutes the first step towards HIV treatment and prevention services. HIV self‐testing (HIVST), whereby individuals collect their own specimen, conduct their own test and interpret the results, allows individuals to learn their HIV status conveniently and privately, as well as to decide when and where to attend post‐test services. Accurate estimation of the proportion of those tested who link to additional HIV care, treatment and prevention services is critical in quantifying the health impact of HIV testing. As HIVST becomes integrated into testing programmes worldwide, implementers in diverse settings will need to measure the effectiveness of their programmes to ensure self‐testers link to onward care and services. This can be challenging, and community health programmes in many contexts find it difficult to track referral uptake and equity 1.

We draw upon experience from the Self‐Testing in AfRica (STAR) Initiative in 2015 to 2017 to identify three lessons for measurement of linkage following HIVST. In STAR, two pragmatic cluster‐randomized trials evaluated the effectiveness of continuous HIVST distribution over 12 months in increasing testing coverage and linkage to care in Malawi and Zambia. A third trial, in Zimbabwe, evaluated the effectiveness of an incentive to promote linkage following a short, campaign‐style HIVST distribution programme, and included a non‐randomized component assessing the association between HIVST distribution and antiretroviral therapy (ART) initiations in nearby clinics. Details are provided elsewhere 2, 3. Each trial incorporated a household survey and data collection from health facilities to evaluate changes in HIV testing coverage and linkage to confirmatory testing, care and prevention (Table 1).

**Table 1:** STAR initiative trials and confirmatory testing and linkage to care and prevention measurements

Trial location	Trial research questions	Primary and secondary trial outcomes	Household survey linkage questions	Clinic data linkage measures
Malawi 10	What is the impact of community‐based HIVST distribution over 12 months on HIV testing and ART initiation?	**Primary:** HIV testing within 12 months of start of HIVST distribution **Secondary:** Lifetime HIV testing, ART initiation	**Confirmatory testing and HIV care:** After each of last three HIV tests, if test was reactive: After this HIV test, did you receive a test confirming your HIV diagnosis or additional care related to your HIV status? If yes, what care did you receive? ○ Follow‐up test to confirm results of this test ○ Start ART for the first time ○ Restarted on ART ○ Other care (not including either confirmatory test or ART) **VMMC:** After each of last three HIV tests, if test was non‐reactive or no result reported, and respondent was male: Did you go for VMMC after this test?	During 12 months of HIVST distribution: ART initiations and HTS by month for 12 months using clinic data ART initiations among clients reporting HIVST use
Zambia 11	What is the impact of the community‐based distribution of HIVST over 12 months on recent HIV testing?	**Primary**: HIV testing within 12 months prior to endline survey **Secondary**: Lifetime HIV testing, ART initiation, ART retention, uptake of VMMC services among men		Zambia study also collected data on HTS by month.
Zimbabwe 12	Do financial incentives for distributors in two‐month HIVST campaigns improve timely linkage among clients? Do community‐based HIVST campaigns increased facility‐based ART initiation?	**Primary**: Attendance at any care service **Secondary**: Self‐test uptake, uptake of PSI outreach services, uptake of VMMC among men, uptake of confirmatory testing among respondents with reactive HIVST, uptake of ART among respondents with reactive HIVST, uptake of self‐tests among people on ART	**Confirmatory testing:** Did you have an HIV test in order to confirm the results you obtained during self‐testing? **HIV care and VMMC**: Since about six weeks ago (when community‐based distributors came to your area to distribute self‐test kits), have you been to a clinic or HIV testing facility for any service that you wanted for yourself? If yes, what services did you seek? HIV counselling and testing Treatment for an ailment I was suffering from Filling of my regular prescription/completing my regular medical check‐up HIV counselling only CD4 testing Family planning services TB screening Blood pressure checks Cervical cancer screening Voluntary medical male circumcision Biomarkers for measuring HIV status, viral load, recency of infection and ART use collected	Count of ART initiations per clinic data at government clinics in districts where HIVST occurred, including data from six months prior to distribution campaign and three months after distribution campaign

ART, antiretroviral therapy; HIVST, HIV self‐testing; HTS, HIV testing services; PSI, Population Services International; VMMC, voluntary medical male circumcision.

## Lesson 1. Collect usage information from users, not just from distributors and clinics

User household surveys and qualitative research are typically more time‐consuming to collect than routine data from distributors and clinics. However, this type of data is vital for understanding how self‐tests are being used. For example, in Zimbabwe, 289 survey respondents reported a reactive HIVST, of whom 216 (75%) were already on ART 2. Formative qualitative work indicated that individuals already known to be positive self‐tested in trial communities to avoid inadvertent disclosure, to confirm their status or check for cure. Without accounting for unintended use of HIVST by persons already diagnosed and linked into HIV care, the number of HIVSTs used by undiagnosed individuals will be overestimated, and both linkage and cost‐effectiveness measures are likely to be overestimated.

## Lesson 2. The quality of linkage measures using clinic data depend not only on the quality of clinic data, but also on the self‐testing distribution model and local context

In two STAR trials, we had contrasting findings about the effectiveness of self‐test distribution on increasing ART initiations at local clinics, measured by routine clinic data. In Malawi, clinics in areas with self‐test distribution had a 14% increase in ART initiations compared with clinics in areas without HIVST distribution (adjusted risk ratio (aRR) = 1.14; 95% CI: 0.75 to 1.75) 4, but the wide confidence intervals suggest limited statistical evidence for effectiveness. In contrast, the study in Zimbabwe showed a somewhat larger increase in local ART initiations in areas with HIVST distribution with stronger evidence for effectiveness (aRR=1.27; 95% CI: 1.14 to 1.43) 5.

These studies used different designs, and the trial in Zimbabwe had more clusters receiving HIVST distribution and more power to detect differences. However, we believe these estimates may also have been driven by the context and the duration of distribution. Universal test‐and‐treat interventions, including the HPTN 071 (PopART) and ANRS 12249 trials, have found that linkage to care remains low even after substantial efforts supporting testing and linkage. This suggests that it is necessary to consider how contextual factors apart from the availability of HIV testing services impact linkage to care 6, 7. The Zimbabwe study used a shorter, campaign approach to distributing HIVST and the implementing organization (Population Services International) had initiatives facilitating linkage‐to‐care across the study area. In contrast, distribution in Malawi was continuous over 12 months, with linkage more diffuse across an extended period. It is likely easier to detect increased linkage at facilities if HIVST kits are distributed in short concentrated periods and in contexts where linkage after testing is relatively easy for users. For programmes working in areas or in populations where linkage is difficult, or for those distributing HIVST over an extended period, alternative methods of measuring linkage may provide more power to detect effects.

## Lesson 3. Measure both linkage to treatment and to prevention

Increasing the availability and uptake of HIV prevention services is increasingly recognized as necessary for reducing HIV incidence rates 8. As testing and treatment expand, the number of new diagnoses identified by testing programmes is likely to decrease, and consequently, the denominators of linkage‐to‐care measures will shrink. Expanding to include linkage‐to‐prevention of persons not infected with HIV will increase power to detect impacts of HIVST on population health.

In the STAR trials described above, linkage to prevention was a consideration, but not the focus. For example, in Zambia, voluntary medical male circumcision (VMMC) mobilizers affiliated with HIVST intervention clinics received HIVST kits to distribute to clients. However, only four of the six intervention clinics had affiliated VMMC mobilizers and the proportion of test kits distributed within each area by VMMC mobilizers was low overall (<1% to 11% across areas). There was a positive association between the intervention and self‐reported VMMC uptake, but this was not statistically significant (aRR: 1.36; 95% CI: 0.49 to 3.78) 5. With collaborators, STAR is now undertaking trials to assess whether HIVST can be used to generate demand for prevention services, including pre‐exposure prophylaxis (PrEP) among adolescent girls and young women in KwaZulu‐Natal, South Africa 9 and VMMC among men in urban Zimbabwe.

To summarize, defining and estimating linkage following HIVST is complex, and there is no single measurement strategy that will fit the needs of all HIVST implementers. Researchers and implementers must account for contextual factors that may affect users’ likelihood of linking and the amount of time required to link when developing strategies to measure linkage following HIVST. In STAR studies, it was difficult to anticipate who would use HIVST, and where and when self‐testers would link to care. Where possible, collecting data on users’ self‐testing and linkage experiences can provide valuable insight.

## Competing interests

The authors have no competing interests.

## Authors’ contributions

MN, HAW and KF conceptualized the paper. MN wrote the first draft. All authors provided comments on the paper.
